# A novel skin-stretching device for closing large skin-soft tissue defects after soft tissue sarcoma resection

**DOI:** 10.1186/s12957-020-02022-3

**Published:** 2020-09-17

**Authors:** Qiang Wu, Zengwu Shao, Yubin Li, Saroj Rai, Min Cui, Ying Yang, Baichuan Wang

**Affiliations:** 1 Department of Orthopedics, Union Hospital, Tongji Medical College, Huazhong University of Science and Technology, Wuhan, 430022 China; 2 Department of Orthopedics, Linqing City people’s Hospital, Linqing, 252600 Shandong China; 3 National Trauma Center, National Academy of Medical Sciences, Kathmandu, Nepal; 4 Department of Operation, Union Hospital, Tongji Medical College, Huazhong University of Science and Technology, Wuhan, 430022 China

**Keywords:** Soft tissue sarcoma, Radical resection, Tissue expansion, Tension suture, Complications

## Abstract

**Background:**

Closure of large skin-soft tissue defects following soft tissue sarcoma (STS) resection has been a great challenge. The objective of this study was to evaluate the effectiveness of a novel, simple, and cheap skin-stretching device (bidirectional regulation-hook skin closure system, BHS) for closing large skin-soft tissue defects resulting from the removal of STS and the complications associated with the use of the BHS.

**Methods:**

From January 2017 to September 2018, 25 patients with STS underwent BHS therapy after tumor resection. BHS was used for two main clinical applications: securing wound closure after high-tension suture closure and delayed wound closure. We described a detailed reconstruction procedure regarding this therapy. Wound closure and complications associated with BHS therapy were recorded. We also analyzed tumor recurrence and metastases.

**Results:**

All patients were observed for 16–36 months with an average follow-up of 25.6 months. During the follow-up period, no significant functional restriction was observed and the final scar was aesthetically acceptable. Superficial wound infection occurred in six patients, wound edge ischemia in two patients, and small skin tears in two patients. Two patients developed pulmonary metastasis, two patients had a local recurrence, and one patient died of pulmonary metastasis.

**Conclusions:**

BHS therapy can effectively close large skin-soft tissue defects following STS resection and obtain acceptable functional results, without severe complications. However, larger studies are required to further evaluate the effectiveness, indications, and complications of BHS therapy.

## Introduction

Soft tissue sarcoma (STS) is an uncommon malignant tumor that originates from primitive mesenchymal tissue. At present, treatment of STS is a multimodal therapy that involves surgical resection, chemotherapy, and radiotherapy [1]. Among these, surgical resection is the mainstay of therapy. However, it frequently results in large skin-soft tissue defect and exposure of vital structures because of the wide margins that need to be taken.

Closure of large skin-soft tissue defects following STS resection is a common reconstructive challenge. Primary closure of wounds with conventional suturing techniques is usually the optimal solution because of its simplicity and acceptable outcomes, yet it is often limited, particularly with regard to large wounds [2]. The most commonly used methods for closing large skin-soft tissue defects following STS resection are local or regional flaps and free tissue flaps [1, 3–6]. However, these methods are often associated with relatively complex surgical procedures, long operation time, significant donor site morbidity, extended hospitalization, and increased costs. In recent years, various external skin-stretching devices, as a promising alternative, have been used to close different types of large skin-soft tissue defects, and acceptable results had been obtained [7–15]. These types of devices take full advantage of viscoelastic properties of the skin (such as mechanical creep and stress relaxation) [16–18] to provide immediate or delayed primary wound closure while avoiding the shortcomings of the aforementioned techniques. However, the application of many devices may be greatly restricted due to a variety of reasons such as different countries and high cost. In this study, we introduced a novel, simple, and cheap skin-stretching device (bidirectional regulation-hook skin closure system, also called BHS; Tianjin Xinzhong Medical Devices CO., LTD, China) to close large skin-soft tissue defects following STS resection.

The objective of this study was to evaluate the effectiveness of the novel skin-stretching device BHS for closing large skin-soft tissue defects resulting from the removal of STS. Moreover, we also evaluated the complications associated with the use of the device, and tumor recurrence and metastases.

## Patients and methods

### Clinical data

From January 2017 to September 2018, following approval by our hospital ethical committee, 25 patients who underwent a novel skin-stretching device (BHS) therapy after STS resection were enrolled in this study. All participating patients gave written informed consent. Inclusion criteria were an open skin defect wound on the limbs or trunk in which direct closure was impossible or primary closure was achieved but with a high tension. Patients with severe cardiopulmonary diseases, vascular diseases, or uncontrolled diabetes mellitus; patients with damaged skin (e.g., severe edema skin and deeply scarred wound edges); and patients refusing the procedure were excluded from this study. BHS was used for two main clinical applications: (a) securing wound closure after high-tension suture closure and (b) delayed wound closure.

The pathological diagnoses of 20 patients were obtained by preoperative needle biopsy, while those of the other 5 patients were obtained by excisional biopsy (done in other institutions). Nine patients were diagnosed with fibrosarcoma, 5 with synovial sarcoma, 3 with undifferentiated sarcoma, 3 with melanoma, 2 with skin protuberant fibrosarcoma, 2 with clear cell sarcoma, and 1 with leiomyosarcoma. The five patients with excisional biopsy had to undergo secondary wide resection due to positive resection margins.

Viscoelastic properties of the skin vary in different sites, and therefore, the site, width, and length of the wound are critical for successful wound closure. These wound parameters were recorded in detail. In addition, information including postoperative wound dressing, skin stretching, wound closure, and follow-up were also recorded (Table 1). The scar tissues were assessed by the following aspects: color, thickness, relief, pliability, and surface area [19].

**Table 1:** Summary data for all patients

Variables	Value
**Biopsy (*n*, %)**	
Needle biopsy	20 (80%)
Excisional biopsy	5 (20%)
**Pathological diagnoses (*n*, %)**	
Fibrosarcoma	9 (36%)
Synovial sarcoma	5 (20%)
Undifferentiated sarcoma	3 (12%)
Melanoma,	3 (12%)
Skin protuberant fibrosarcoma	2 (8%)
Clear cell sarcoma	2 (8%)
Leiomyosarcoma	1 (4%)
**Wound location (*n*, %)**	
Upper extremity	Forearm 3 (12%); upper arm 3 (12%)
Lower extremity	Thigh 8 (32%), calf 6 (24%), feet 2 (8%)
Trunk	Back 2 (8%), abdomen 1 (4%)
**Wound size (cm)**	
Length: range, mean (SD) cm	3.5–19, 10.1 (4.1)
Width: range, mean (SD) cm	1.5–11, 6.1 (2.9)
**Skin stretching (*n*, %)**	
Intraoperative stretching	7 (28%)
Postoperative stretching	18 (72%)
**Time to start postoperative chemo and/or radio**	
**Range, mean (SD) days**	18–31, 23.2 (4.5)
**Follow-up time: range, mean (SD) months**	16-36, 25.6 (3.7)
**Complications (n, %)**	
Minor superficial wound infection	6 (24%)
Wound edge ischemia	2 (8%)
Small skin tears	2 (8%)
**Survival status (*n*, %)**	
DFS	20 (80%)
AWD	4 (16%; 2 with pulmonary metastasis, 2 with local recurrence)
DOD	1 (4%)

*SD* standard deviation, *DF*S disease-free survival, *AWD* alive with disease, *DOD* died of disease

### Skin-stretching device

**Fig. 1:**
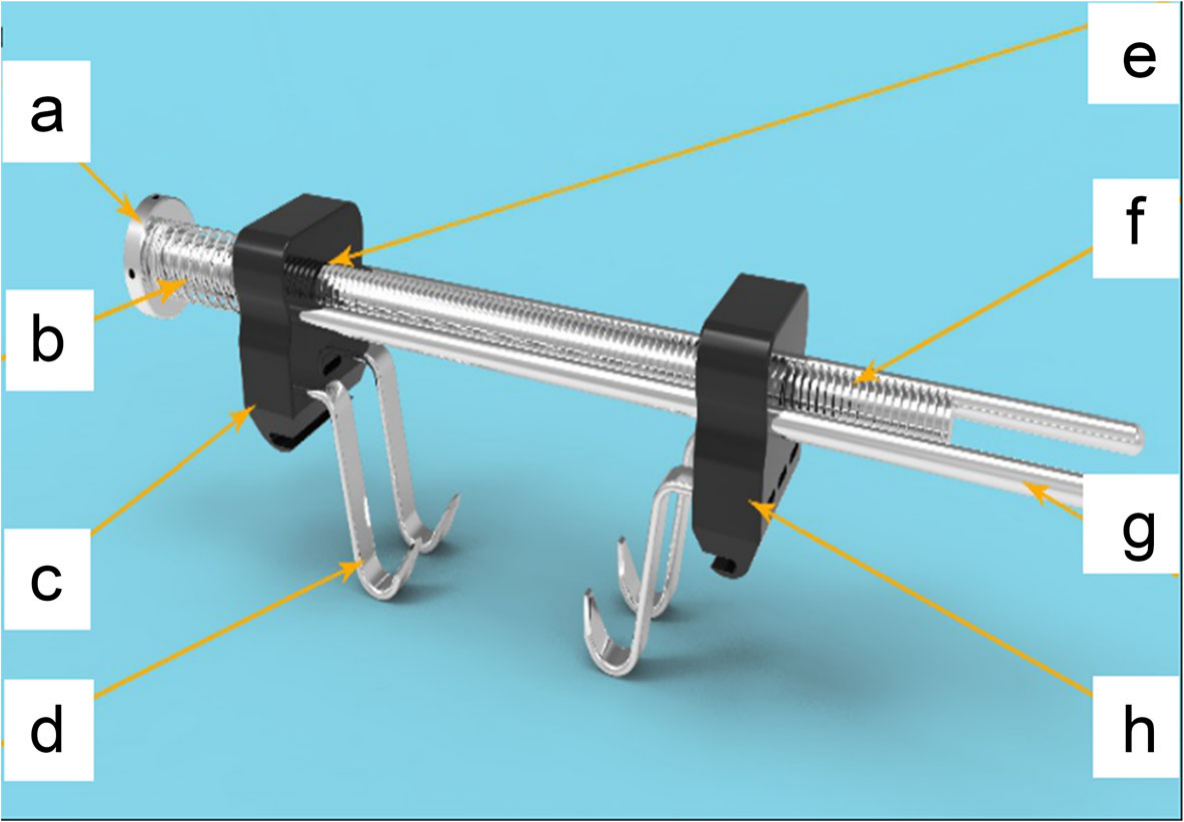
The novel skin-stretching device, BHS. **a** Threaded rod cap, **b** spring, **c** hook holder module, **d** sharp hook, **e** threaded plunger hole, **f** threaded rod, and (**g**) guide rod. The two hook holder modules can be approximated together by rotating the threaded rod and pushing the spring along the guide rods

BHS is composed of two hook holder modules and a compression module. The hook holder module is made of polyetheretherketone (PEEK) material, and the compression module is made of stainless steel. Each holder module contains a threaded plunger hole, two guide holes, and three hook holder holes with sharp hooks at their tips facing each other. The compression module is formed of a threaded rod, a spring, and two guide rods. The threaded rod is passed through the threaded plunger holes on the hook holder modules with the aid of the two guide rods. The two hook holder modules can be approximated together by rotating the threaded rod and pushing the spring along the guide rods (Fig. 1). The price of each BHS device is about $ 80.

### Surgical technique

**Fig. 2:**
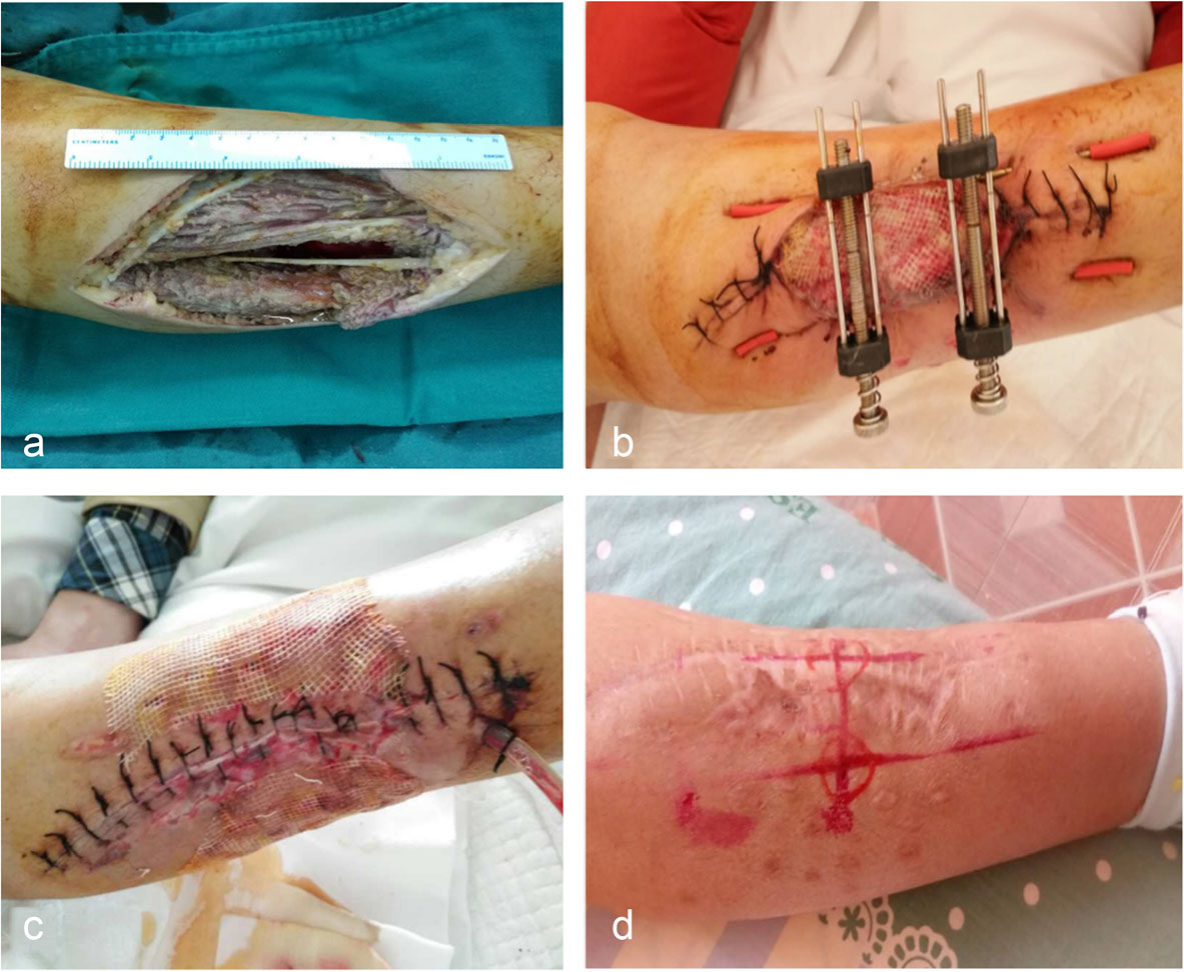
Reconstruction of a large skin-soft tissue defect in the right calf by using BHS. **a** Wound after fibrosarcoma resection measuring about 13 × 8 cm. **b** Gradual approximation of wound edges. **c** Wound was closed 8 days after gradual stretching. **d** Three months following reconstruction, uneventful recovery, with acceptable aesthetic result and minor widening of the scar

**Fig. 3:**
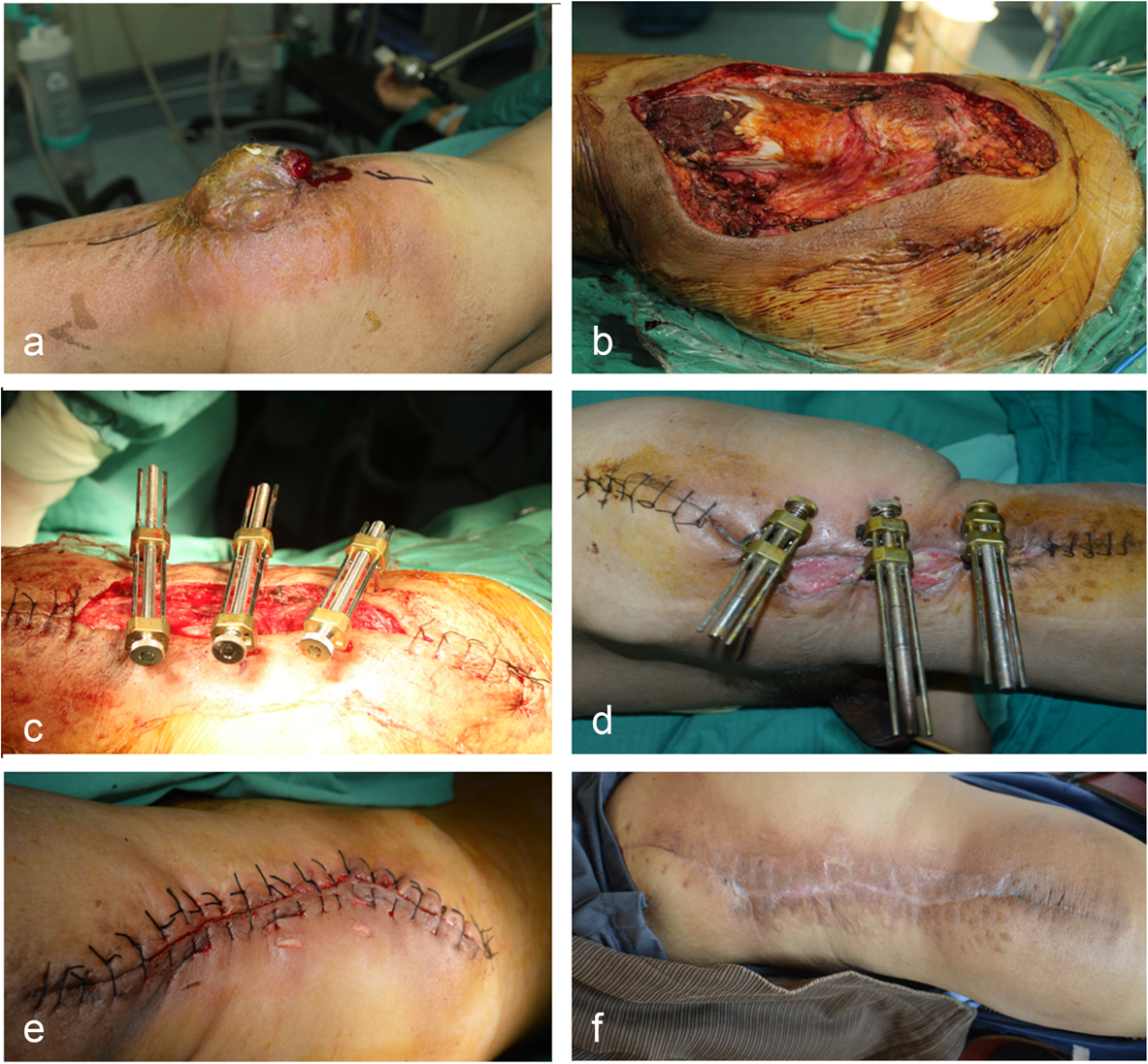
Reconstruction of a large skin-soft tissue defect in the right thigh by using BHS. **a** Recurred fibrosarcoma in the right thigh. **b** Wound after tumor resection measuring about 19 × 11 cm. **c**, **d** Gradual approximation of wound edges. **e** Wound was sutured 12 days after gradual stretching. **f** Eighteen months following reconstruction, the photograph showed acceptable aesthetic result and mild scar

After complete removal of the tumor in accordance with the principle of STS resection, large skin-soft tissue defects were treated by using BHS. After cleaning the wound, 2.0-mm diameter Kirschner wires (KWs) were inserted into the skin and subcutaneous tissue approximately 1 cm away from wound margins. They passed subcutaneously for some distance, and then out of the skin to form two or more equal-sized bridges as required. Then, the two hook holder modules were placed on the KWs bridges (or the skin and subcutaneous tissue directly) by using hooks. The threaded rod was passed through the threaded plunger hole and gently advanced until the wound begins to resist closure. In order to avoid overstretching of the skin, the color of the wound edges was carefully observed during the period of gradual approximation. For wounds with immediate closure under high tension, the device was still in place until the stretching force significantly reduced. For wounds with delayed closure, the wound edges were approximated one to two times 1 day by the patient him/herself. The device was removed when the wound edges were completely together, and then the wound was sutured (Figs. 2 and 3). The number of skin-stretching devices required depended on the size of the wound.

### Postoperative treatments

Patients with open wounds received an intravenous injection of broad-spectrum antibiotic both preoperatively and postoperatively. For wounds with immediate closure, conventional dressing change was performed every 2 days until the wound healed completely. For wounds with delayed closure, the open wounds were covered with vaseline gauze and silver ion surgical dressing, and the dressings were changed every 2 days. In order to avoid the necrosis and laceration of the skin, the color of the wound edge was carefully observed and the status of pain was assessed during the progress of gradual approximation. Wound biopsy for tissue culture was performed to evaluate wound infection before suturing. Once the wound was sutured, patient follow-up took place at 7, 14, and 21 days and every 3 months thereafter. All patients received postoperative chemotherapy and/or radiotherapy or biological immunization therapy after wound healing.

## Results

There were 15 male and 10 female patients, and their average age was 56.1 years (range, 25–71 years). The rate of wound closure was determined according to the size of the defect. Of the 25 patients, 7 patients underwent immediate intraoperative wound closure with the aid of BHS and 18 patients underwent postoperative gradual approximation to secure wound closure (Table 1). For wounds with delayed closure, the average time required for wound closure was 7.8 days (range, 5–15 days). The average time to start postoperative chemotherapy and/or radiotherapy is 23.2 days (range, 18–31 days).

The mean follow-up time was 25.6 months (range, 16–36 months). Twenty patients were alive without evidence of disease, 2 patients with fibrosarcoma were alive with local recurrence and had to undergo secondary resection, 2 patients with synovial sarcoma and fibrosarcoma were alive with pulmonary metastasis, and 1 patient with fibrosarcoma died of pulmonary metastasis 25 months after surgery. All patients showed scarring according to the length of the wound. Two patients complained of mild pain and itching at the time of final follow-up. In all patients, no significant functional restriction of adjacent joints was observed and the final scars were aesthetically acceptable.

None of our patients experienced severe complications in this study. Six of 18 patients with delayed wound closure developed minor superficial wound infection. However, these wounds were closed directly after thorough debridement and completely healed after 2 weeks of dressing change. Wound edge ischemia occurred in 2 patients with immediate wound closure, which was successfully treated by adjusting the threaded rod and/or removing some sutures to reduce the tension of the wound edge. Small skin tears surrounding the wound occurred in 2 patients with the hooks directly hooked on the skin. However, none of them required any secondary surgical procedure for these small skin tears.

## Discussion

STS is uncommon malignant tumor accounting for approximately 1% of all malignancies [20, 21]. Radical surgical resection is the most important approach in the curative multimodal therapy of STS [1, 20]. However, large skin-soft tissue defect coverage following tumor resection presents a challenge for reconstructive surgeons, especially for surgeons with the absence of reconstructive expertise.

Closure of large skin-soft tissue defects following tumor resection as well as the closure of large trauma or chronic wounds could often not be achieved by conventional suturing technique due to high tension [2]. The difference is that reconstruction of skin-soft tissue defects following tumor resection not only requires consideration of functional and aesthetic aspects, but also oncological factors [8, 10, 22]. Currently, many surgical methods such as skin graft, local flap, free tissue transfer procedure, tissue expansion, or various combinations of the aforementioned options have been used to close these large defects following tumor resection [6–8, 10–13, 22–26]. Of these methods, tissue expansion procedure may be a promising, cost-effective treatment option [22, 24–26]. It significantly downgrades surgical complexity, reduces operating time, and shortens hospital stay. Although internal tissue expansion technique has been widely used in the cosmetology plastic surgery and obtained good clinical outcomes [27, 28], it may be greatly limited in the treatment of soft-tissue tumors, especially malignant tumors, due to long expansion period and potential stimulating effects on tumors.

Recently, external tissue expansion technique was developed with the intention to close large skin-soft tissue defects that cannot be sutured directly, reduce preoperative preparation time, and decrease complications. In 1993, Hirschowitz et al. firstly described an external skin-stretching device which harnesses the viscoelastic properties of the skin to close open wounds [13]. Since then, many modifications, such as SureClosure®, Wiseband®, and TopClosure®, have been introduced and reported by different authors and obtained acceptable outcomes [7–10, 12, 15, 25, 29] (Table 2). However, due to geographical, economical, or other special factors, many of them are not used in many hospitals or countries. To expand the application of this technique, we here introduced a novel, simple, and cheap skin-stretching device to close large skin-soft tissue defects following STS resection and evaluated its efficacy and safety as well as the related complications.

**Table 2:** Representative skin-stretching devices for the closure of large skin-soft tissue wounds

Study	Devices	No. of wounds	Aetiology of the wounds	Manner of wound closure	Follow-up (months)	Complications
Kanjoor et al. [7]	SureClosure	4	Trauma (50%), burns (50%)	Immediate closure, 2 Delayed closure, 2	24; 12; 12; NC	Minimal scar widening, 1; compartment syndrome, 1
Barnea et al. [10]	Wisebands device	22	Trauma (35%), surgery (35%), tumors (25%), burns (5%)	Immediate closure, 6 Delayed closure, 16	12 (mean)	Intractable pain, 1; serious wound infection, 1
Ismavel et al. [30]	Kirschner wires	10	Trauma (40%), fasciotomy wounds (30%), surgery (30%)	Delayed closure, 10	9.9 (mean)	Wound infection, 4; partial wire cutout, 3; partial wound dehiscence, 2
Santiago et al. [9]	DermaClose RC	14	Blast-related injury	Delayed closure, 12	NC	Blistering, 2; maceration of the wound edges, 3
Verhaegen et al. [15]	Skin Stretch system	8	Burns	Immediate closure, 8	12.1 (mean)	Wound infection, 2
Topaz et al. [8]	TopClosure® TRS	3	Malignant soft tissue tumors	Immediate closure, 3	6; 10; 18	Wound dehiscence, 1; blistering, 1; Scar widening, 1; limitation in ROM, 1
Aboelatta et al. [31]	Home-made wound approximation device	34	Posttraumatic alopecia or ulcers (79%); post burn scars (21%)	Immediate closure, 25 Delayed closure, 9	Over a period of 48 months	Wound infection, 21; wound dehiscence, 6; edge necrosis, 2; pressure necrosis, 1; adherent scars, 6
Karkos et al. [32]	ETE Blomqvist	8	Trauma	Delayed closure, 8	Over a period of 144 months	Wound infection, 1
Current study	BHS device	25	Malignant soft tissue tumors	Immediate closure, 7 Delayed closure, 18	25.6 (mean)	Superficial wound infection, 6; wound edge ischemia, 2; small skin tears, 2.

*NC* not clear, *ROM* range of movement, *ETE* external tissue extender, *BHS* bidirectional regulation-hook skin closure system

Compared with many previous skin-stretching devices, the novel BHS device has many significant advantages: (a) it has a very simple design and very cheap; (b) it is made of PEEK material and stainless steel that can be sterilized and therefore can be reused; (c) it can be regulated bidirectionally, which not only ensures the closure of large skin-soft tissue wound, but also maintains appropriate tension at the edge of the wound; (d) the assisted use of KWs along the entire length of the wound edge ensures relatively uniform distribution of the stretching force across the wound edge. In addition, it is an easy-to-use device and could be applied under local anesthesia in the out-patient department in most patients with delayed wound approximation (e.g., closure of large chronic wounds), although more clinical evidence of BHS therapy is still needed.

Our results found that the novel BHS device provided the phenomenal ability of stretching the skin for immediate or delayed primary closure of large skin-soft tissue defects following STS resection. As a topical tension-relief platform, the BHS device alleviates the typical tearing and scarring, traditionally inflicted by tension sutures. In our cases, BHS therapy provides acceptable functional and cosmetic wound closure, which is comparable with many previous results [8, 31]. We would like to emphasize that undermining of the skin edges and adjacent tissues is not required for successful execution of wound closure with this method. Although undermining can provide a small additional tension decrease [13], it may result in dead space, hematoma, and skin edge necrosis.

Wound dehiscence, wound edge necrosis, and skin tears are the most reported complications [14, 30, 31] (Table 2). Studies showed that the wound approximation technique was associated with relatively high wound dehiscence (20–33%) [8, 30, 31]. However, no patient developed wound dehiscence in this small series. This might be related with the difference in defect size, wound edge tension, stretching time, and time of suture removal in different studies. Although no wound necrosis was observed in our patients, wound edge ischemia occurred in 2 patients with immediate wound closure. They were successfully treated by adjusting the threaded rod and removing some sutures to reduce the tension of the wound edge. Skin tears occurred in 2 patients with the hooks directly hooked on the skin. However, the similar problems did not arise when the KWs were used. Of note, local infection is a relatively rare complication [7–10, 32]. However, 6 patients were diagnosed with minor superficial wound infection in this study. This may be greatly attributed to the fact that some patients in our study had infected wounds or vaseline gauze alone was used at the early stage. On the whole, the expertise and experience of the surgeons are critical to reduce these complications.

Admittedly, this study has some limitations. The main limitation is that it had no appropriately matched control group. Another limitation of this study is that this device was only used for closure of large skin-soft tissue defects following STS resection in the present study. Hence, the indications and contraindications for BHS therapy are still unclear. A wider scope of application (such as traumatic and chronic wounds) is required to further evaluate this technique. Finally, it is limited by the small total number of patients included. More cases need to be studied before widely using this device.

## Conclusion

In this study, we introduced a novel, simple, and cheap skin-stretching device BHS and presented our experiences using BHS therapy. The device is technically simple to apply and permits bidirectional control depending on the situation of the wound edges. BHS therapy facilitates the closure of large skin-soft tissue defects following STS resection, and acceptable functional and cosmetic results are obtained, without severe complications. In summary, BHS provides the surgeon an important tool for the closure of large skin-soft tissue defects, although a larger scale remains necessary to evaluate the effectiveness, indications, and complications of BHS therapy.

## Data Availability

The data that support the findings of this study are available from the corresponding author upon reasonable request. The data are not publicly available due to privacy or ethical restrictions.

## Abbreviations

STS: Soft tissue sarcoma
BHS: Bidirectional regulation-hook skin closure system
KWs: Kirschner wires
